# Neuroglobin correlates with cryptochrome-1 in obstructive sleep apnea with primary aldosteronism

**DOI:** 10.1371/journal.pone.0204390

**Published:** 2018-09-20

**Authors:** Rimawati Tedjasukmana, Jan Sudir Purba, Septelia Inawati Wanandi, Franciscus D. Suyatna

**Affiliations:** 1 Biomedical Department, Universitas Indonesia, Jakarta, Indonesia; 2 Department of Neurology, Universitas Krida Wacana, Jakarta, Indonesia; 3 Department of Neurology, Universitas Indonesia, Jakarta, Indonesia; 4 Department of Biochemistry and Molecular Biology, Universitas Indonesia, Jakarta, Indonesia; 5 Department of Pharmacology, Universitas Indonesia, Jakarta, Indonesia; University of Rome Tor Vergata, ITALY

## Abstract

**Background:**

Neuroglobin (Ngb) is highly expressed in the suprachiasmatic nucleus, and can regulate Per1 gene expression. It is still not known whether Ngb also influences Cryptochrome (Cry). Cry is implicated in hypertension and primary aldosteronism (PA) in mice. There is a strong correlation between Obstructive Sleep Apnea (OSA) and PA. We propose to prove that Ngb and Cry play a role in OSA with PA.

**Methods:**

Subjects were recruited consecutively from residents of Jakarta, Indonesia; subjects aged 30–65 years with moderate to severe OSA and hypertension were included in the study. OSA was diagnosed using an unattended type 2 portable monitor (Alice Pdx), hypertension was diagnosed when morning blood pressure exceeded 140/90 mmHg or when taking anti-hypertensive drugs. Serum concentration of aldosterone, renin, Cry1, Cry2 and Ngb protein were determined using ELISA method. Primary aldosteronism (PA) was defined as ARR ≥20.

**Results:**

Forty subjects were recruited, 26 male and 14 female, median age 52.5 years, BMI 27.46 kg/m^2^, and AHI 34.8 times/hour. We found 16 subjects with PA and 24 non PA. Cry1 and Cry2 did not correlate with ARR in PA and non PA groups. Ngb correlated positively with Cry1 (Spearman’s rho = 0.455, p = 0.038) but not Cry2 in PA patients. Cry1 concentration decreased in severe hypoxia.

**Conclusions:**

Ngb correlates with Cry1 in OSA with PA. There is no correlation between Cry1 or Cry2 with PA

## Introduction

Neuroglobin (Ngb) is a globin with high affinity for oxygen and mostly found in the brain and retina [1]. The physiological function of this protein is not fully understood but discussed with regard to O_2_ supply, the detoxification of reactive oxygen or nitrogen species, and apoptosis protection [2]. It was shown that Ngb is highly expressed in the rat suprachiasmatic nucleus (SCN) [1]. Because of its localization, it is surmised that Ngb plays a role in circadian rhythm. Elimination of Ngb increased Per1 gene expression in the SCN [3]. It is still not known whether Ngb also influences Cryptochrome. Both Cryptochrome and Period proteins play a central role in the inhibitory branch of the autoregulatory transcriptional loop that makes up the clock [4].

Obstructive Sleep Apnea (OSA) and Primary Aldosteronism (PA) commonly coexist in resistant hypertension [5]. However, the cause of increased aldosterone production in OSA is still not known. Doi et al [6] discovered that mice lacking the core clock genes Cryptochrome1 (Cry1) and Cryptochrome2 (Cry2) exhibit primary aldosteronism (PA) and salt-dependent hypertension. Cry1 and Cry2 are required for the normal expression of circadian behavioral rhythms. We surmise that PA in OSA was related with decreased Cry1 and Cry2 expression, which will vindicate Doi’s findings in mice.

Ngb plays a role in circadian rhythm. Hundahl [3] discovered that Ngb influences Per1 gene, perhaps Ngb also influences Cryptochrome. We propose to prove that Ngb and Cry play a role in OSA with PA.

## Materials and methods

### Study participants

The subjects of this study were residents of Jakarta, Indonesia, aged 30 to 65. All subjects provided written informed consent prior to study participation. This study was approved by the Ethics Committee of the Medical Faculty of Universitas Indonesia. Subjects were recruited by home visits. The recruited subjects were diagnosed to have moderate to severe OSA with apnea-hypopnea index (AHI) of 15 or more. The subjects also suffered from hypertension with morning blood pressure 140/90 mmHg or more, or they were already diagnosed as having hypertension and were taking anti-hypertensive medication. Subjects who had tracheostomy or history of upper airway operation, upper airway cancer, congestive heart failure, hypothyroidism, chronic liver failure or were pregnant, were also excluded from this study.

### Blood pressure measurement

Blood pressure was measured manually using a mercury sphygmomanometer and an appropriate-sized cuff after 5 minutes of rest while patients were seated, according to standard guidelines. Blood pressure was measured in the morning after portable polysomnography monitoring.

### OSA screening and diagnosis

Subjects were screened for OSA with Berlin Questionnaire. Subjects with high OSA risk and hypertension underwent polysomnography with type 2 unattended portable monitoring device (Alice Pdx) in their homes. Polysomnography evaluation included airflow monitoring with thermistor and nasal pressure, respiratory effort using piezo belts at the chest and abdominal positions, oxygen saturation using pulse oximetry, heart rate using a 2-lead electrocardiogram, electroencephalogram (C4-M1, C3-M2, O2-M1, O1-M2), submental electromyograms, and bilateral electrooculograms.

Recorded sleep data was scored manually according to standard criteria by an experienced Registered Polysomnographic Technologist. Apnea and hypopnea were defined according to the *AASM Manual for the Scoring of Sleep and Associated Events* [7]. An apnea was defined as complete cessation of airflow for ≥ 10 seconds. Hypopnea was defined as a reduction of airflow ≥30% for at least 10 seconds associated with oxygen desaturation of ≥ 3% or arousal. The apnea-hypopnea index (AHI) was calculated as the total number of apneas plus hypopneas divided by the hours of sleep. OSA was defined as AHI more than 5 times per hour, moderate OSA with AHI 15 to 30 times per hour, and severe OSA with AHI more than 30 times per hour.

### Biochemical evaluation

Blood was drawn in the morning after portable monitoring with subjects in sitting position. Glucose, urea and creatinine serum concentration were measured to rule out diabetes and chronic kidney disease. Aldosterone, renin, Cry1, Cry2, and Ngb serum concentration were determined using ELISA method (Elab Science kits). Primary aldosteronism (PA) was defined as Aldosterone Renin Ratio (ARR) ≥20.

### Statistical evaluation

All values are reported as median (minimum-maximum). Variables were assessed for normality by Shapiro-Wilk test. Values between groups were compared by Mann-Whitney U test or Chi square test where appropriate, also nonparametric Spearman’s correlation test was used, p < 0.05 was considered significant. Data analysis was carried out using SPSS version 23.

## Results

### Subject recruitment

**Fig 1:**
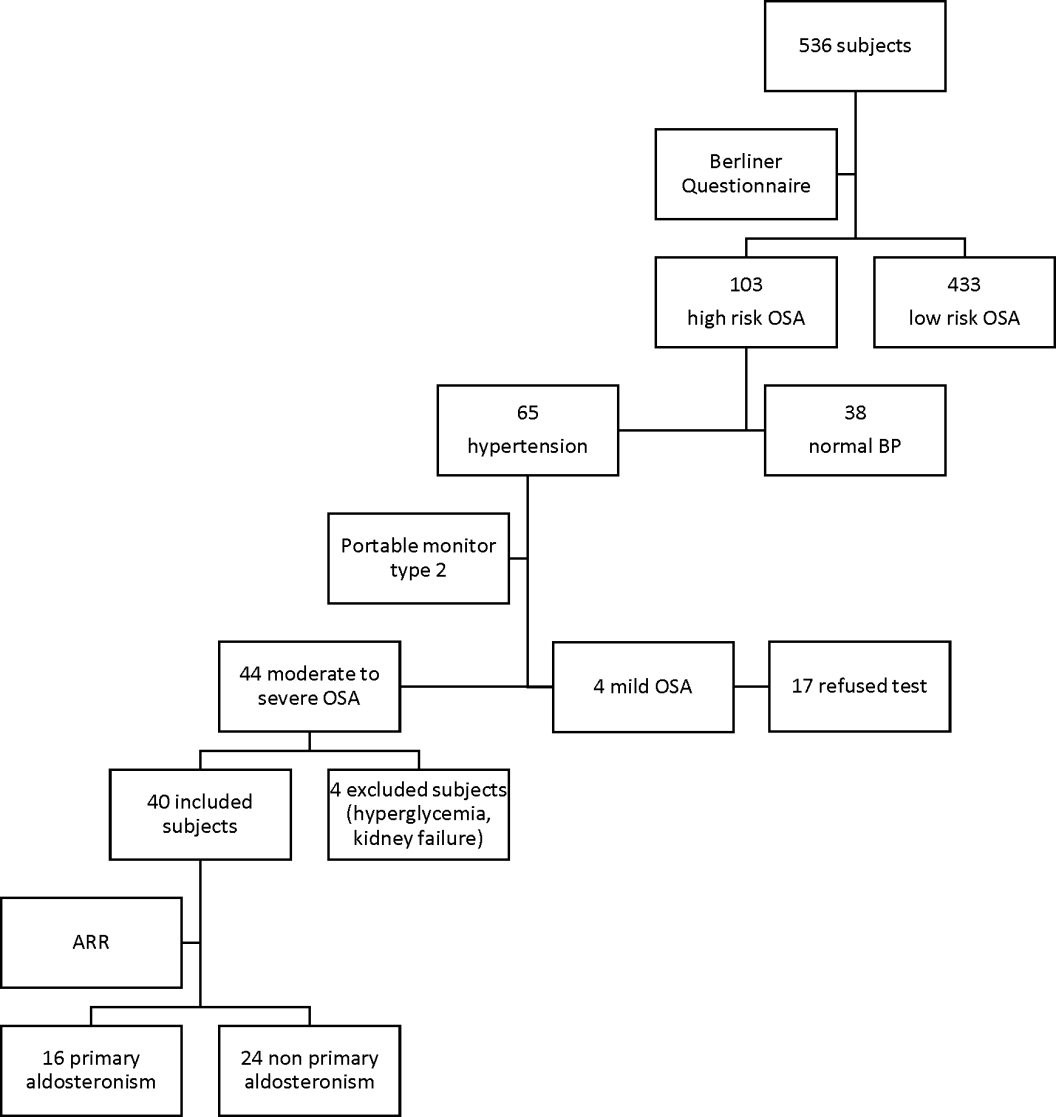
Subject recruitment flowchart.

We randomly select 536 subjects living in 5 districts in the city of Jakarta (population around 10 million), Indonesia, during 2014–2015. Most of the recruited subjects were women (347 subjects/64.7%). All subjects were interviewed using the Indonesian version of the Berlin questionnaire. One hundred and three subjects were identified as high risk for OSA and 65 subjects of this group also had hypertension. After testing with a type 2 portable monitor in the high risk OSA with hypertension group we found 44 subjects with moderate to severe OSA, 4 mild OSA, and 17 subjects refused testing or were unavailable. We enrolled 44 consecutive subjects which concurred with the inclusion criteria. However, four subjects were excluded because they have hyperglycemia (random blood glucose>200 mg/dL), kidney failure (eGFR<60 mL/min/1.73 m^2^), or both. Only 40 subjects were included in this study (**Fig 1**).

### Patient characteristics

There were 40 subjects recruited in this study consisting of 26 male and 14 female subjects. Median age was 52.5 years (minimum 30-maximum 65), BMI 27.46 kg/m^2^ (21.30–34.93), AHI 34.8 times/hour (16.6–99.1), O_2_ nadir 81.50% (20–93). Subjects were classified into PA (primary aldosteronism) and non PA group according to ARR values less or more than 20. We found 16 OSA subjects with PA and 24 non PA subjects (**Table 1**). The two groups were statistically comparable in age, gender, BMI, AHI, O_2_ nadir and renin levels. Only serum aldosterone concentration differentiate these groups. Aldosterone concentration was significantly higher in the PA group with median 7488.56 ng/mL (4217.86–16367.97) compared to non PA group 4687.38 ng/mL (2834.77–15878.67) (p<0.05, Mann Whitney U test). ARR was obviously higher in the PA group with median 35.64 (25.46–532.31) compared to 6.18 (0.80–19.51) in the non PA group.

**Table 1:** Characteristics of OSA subjects with primary aldosteronism (PA) and non PA.

Characteristics	PA	Non PA	*p*
N	16	24	
Age (years)	53 (43–56.5)	52.5 (48.2–59)	0.658^a^
Gender male	13	13	0.079^b^
BMI (kg/m^2^)	27.33 (25.27–31.42)	27.87 (25.65–29.19)	0.847^a^
AHI (times/hour)	41.45 (30.7–57.7)	38.85 (28.6–61.4)	0.934^a^
O_2_ nadir (%)	81 (76.7–85.5)	82.5 (67.5–87)	1.0^a^
Renin (pg/mL)	299.84 (127.39–1367.96)	369.23 (183.9–1377.1)	0.6^a^
Aldosteron (pg/mL)	7488.56 (4849.27–10950.53)	4687.38 (3919.69–6833.73)	<0.05^a^*
ARR	35.64 (25.93–80.15)	6.18 (1.64–12.63)	-
Cry1 (ng/mL)	359.91 (301.45–436.82)	297.72 (252.26–399.03)	0.185^a^
Cry2 (ng/mL)	0.42 (0.12–3.93)	1.07 (0.47–5.99)	0.320^a^
Ngb (ng/mL)	112.45 (49.05–219.86)	128.87 (94.63–205.44)	0.669^a^

Note: values in median (interquartile range/IQR)

^a^ Mann Whitney U test

^b^ Chi square test

*statistically significant

ARR = Aldosterone Renin Ratio, Cry1 = Cryptochrome1, Cry2 = Cryptochrome2, Ngb = Neuroglobin

### Comparison of Cry1 and Cry2 protein level in subjects with primary aldosteronism (PA) and without PA

**Fig 2:**
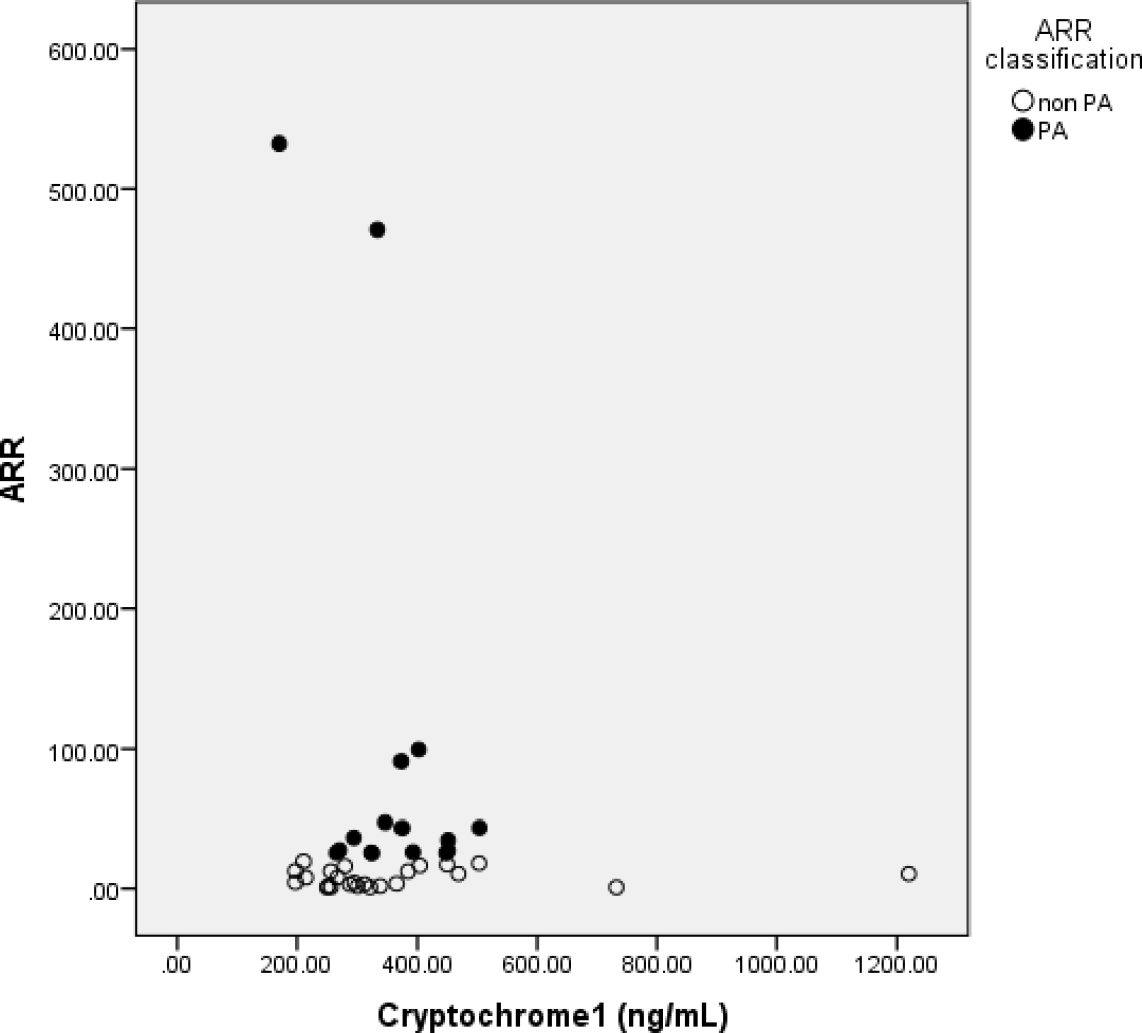
Correlation between Cryptochrome1 serum concentration and Aldosterone Renin Ratio (ARR) in OSA subjects with PA and nonPA. Cryptochrome1 did not correlate with ARR in PA group (Spearman’s rho = -0.015, p = 0.479, n = 16), and also non PA group.

**Fig 3:**
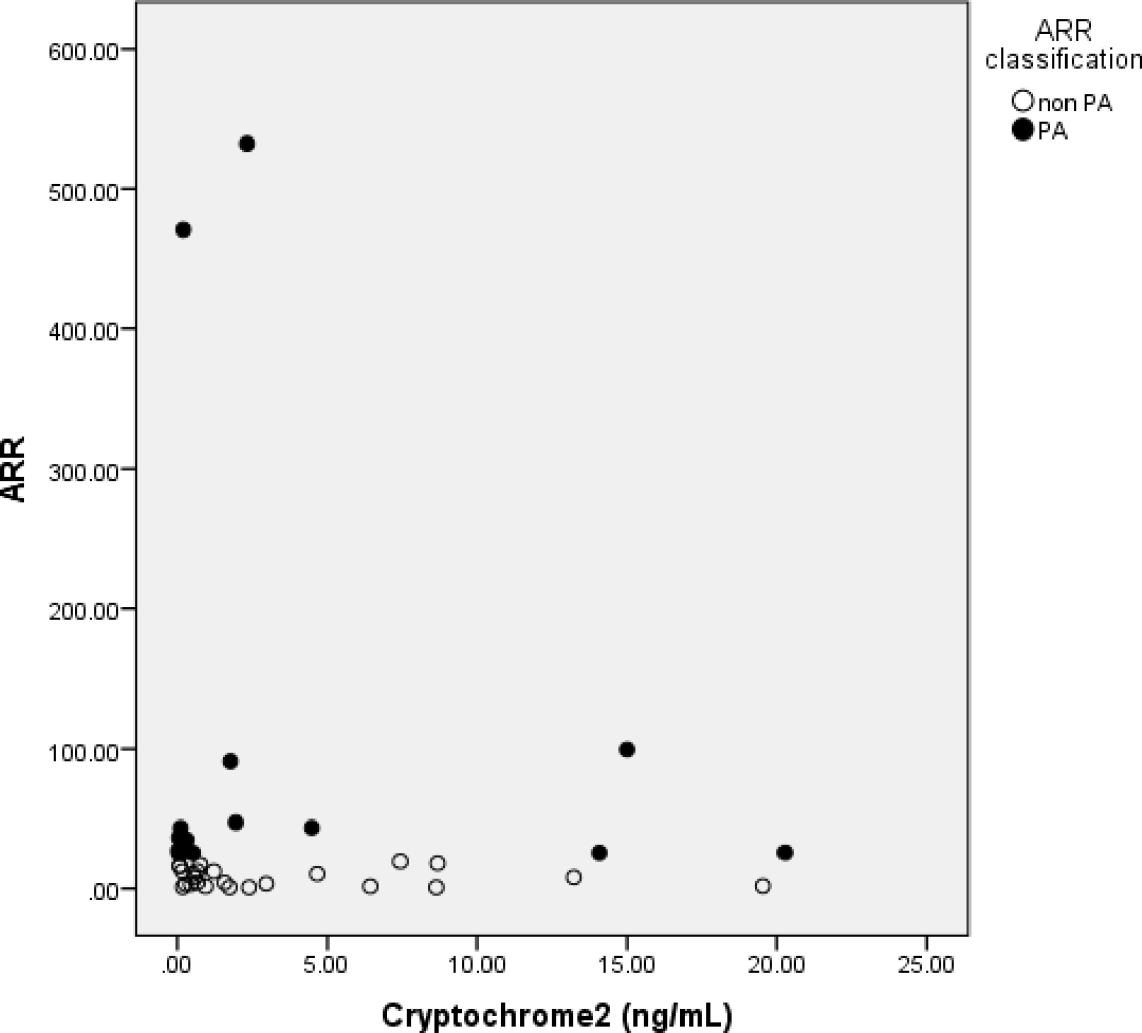
Correlation between Cryptochrome2 serum concentration and Aldosterone Renin Ratio (ARR) in OSA subjects with PA and nonPA. Cryptochrome2 did not correlate with ARR in PA group (Spearman’s rho = -0.015, p = 0.479, n = 16), and also non PA group.

We did not find any significant difference of Cry1 and Cry2 expression in both PA and non PA groups (**Table 1**). We also did not find correlation between Cry1 and ARR in PA (Spearman’s rho = -0.015, p = 0.478, n = 16) or non PA group (Spearman r = 0.065, p = 0.382, n = 24) (**Fig 2**). There was no correlation between Cry2 and ARR in PA (Spearman’s rho r = 0.209, p = 0.219, n = 16) or non PA group (Spearman r = -0.182, p = 0.197, n = 24) (**Fig 3**).

### Relationship between OSA severity, nadir O_2_, Cry1 and Ngb

There was no difference of Cry1 expression in moderate (median 312.26 ng/ml, IQR 255.78–448.47) and severe OSA (median 324.81 ng/ml, IQR 266.07–402.9) (p = 0.881, Mann Whitney U test). However, we found a relationship between Cry1 and nadir O_2_ (p = 0.026, Kruskal Wallis test). In subjects with nadir O_2_ more than 88% median Cry1 concentration was 403.96 ng/mL (388.35–450.03), subjects with nadir O_2_ 60–88% median Cry1 was 322.47 ng/mL (265.54–395.03), and in subjects with nadir O_2_ less than 60% Cry1 was 248.9 ng/mL (210.13–294.13). From these data we conclude that Cry1 concentration decreased in severe hypoxia. We found no difference in Ngb expression between moderate and severe OSA (p = 0.654, Mann = Whitney U test), also there was no relationship between Ngb and nadir O2 (p = 0.469, Kruskal-Wallis test).

### Correlation between Ngb, Cry1 and Cry2 in OSA patients with PA

**Fig 4:**
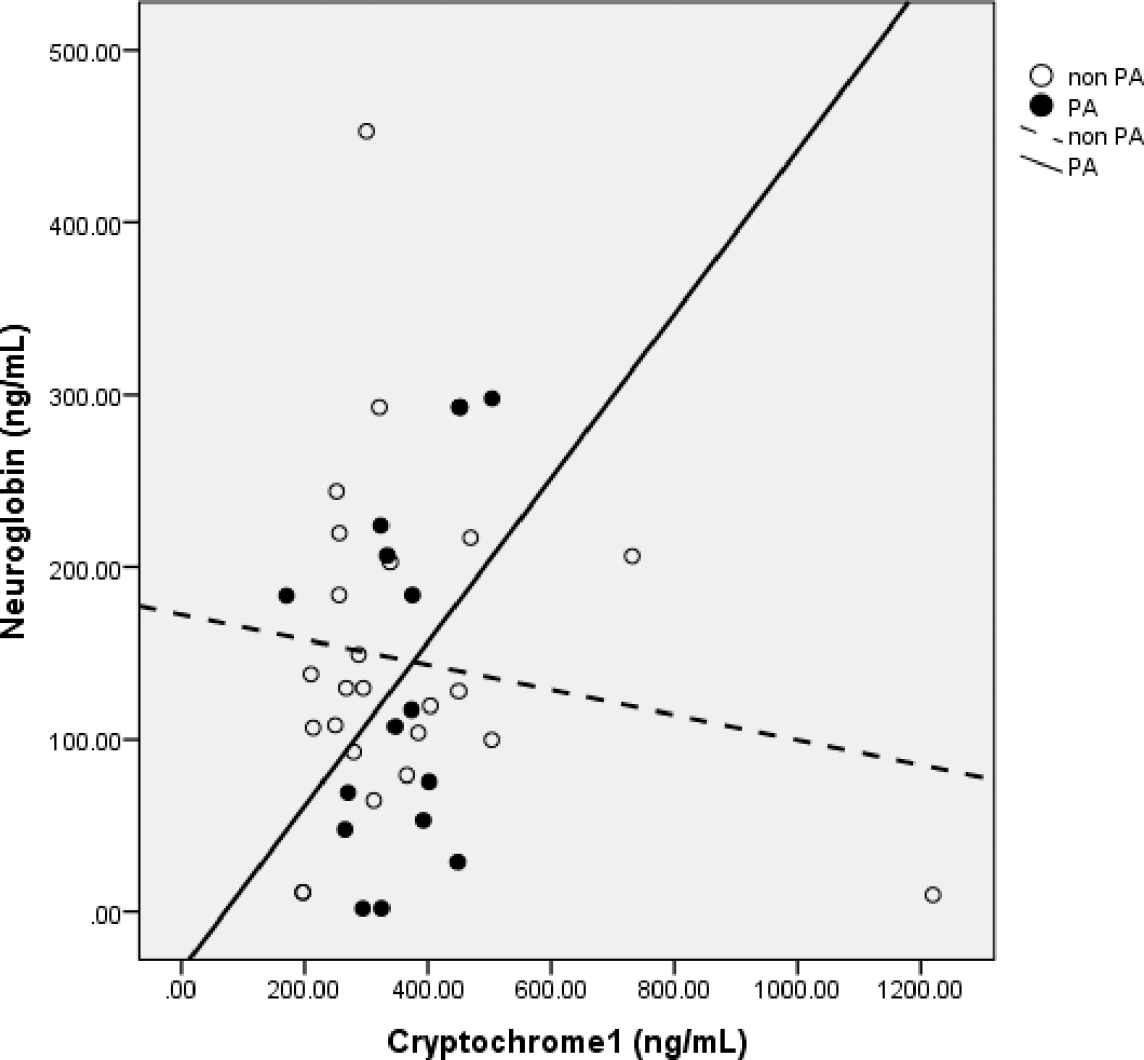
Correlation between Neuroglobin and Cryptochrome1 serum concentration in OSA subjects with PA and non PA. Neuroglobin has positive correlation with Cryptochrome1 in PA group (Spearman’s rho = 0.455, p = 0.038, n = 16), but not in non PA group.

We found positive correlation with moderate power between Ngb and Cry1 in OSA patients with PA (Spearman’s rho = 0.455, p = 0.038, n = 16), but not in the non PA group (Spearman r = 0.036, p = 0.434, n = 24) (**Fig 4**). There was a decrease in Cry1 protein concentration with decreasing Ngb concentration. However, we did not find correlation between Ngb and Cry2 in both PA (Spearman’s rho r = 0.346, p = 0.095, n = 16) and non PA groups (Spearman’s rho = 0.128, p = 0.276, n = 24).

## Discussion

Several studies have discovered an association among resistant hypertension, OSA, and aldosterone [8–10]. However, the cause of the increased prevalence of primary aldosteronism in OSA remains unclear. Aldosterone excess may worsen OSA by promoting accumulation of fluid within the neck, which then contributes to increased upper airway resistance [11]. But perhaps it works both ways, OSA could also cause primary aldosteronism.

In our study 16 (40%) out of 40 subjects with moderate to severe OSA and hypertension also suffer from primary aldosteronism (PA). Aldosterone concentration was significantly higher in the PA group compared to the non PA group. Our subjects were older adults, mostly male, overweight, mostly suffered from severe OSA with low nadir O_2_. However, the PA and non PA groups in our study were similar and comparable except for aldosterone concentration. Increased BMI and OSA severity alone could not explain the higher aldosterone expression in the PA group compared to the non PA group.

In this study we found that Cry1 and Cry2 expressions did not correlate with ARR in OSA patients with and without PA. This finding did not fulfill our hypothesis that Cryptochrome deficiency could increase aldosterone in OSA patients. We also found a positive correlation between Ngb and Cry1 in OSA with PA, but not in the non PA group. No correlation was found between Ngb and Cry2 in both PA and non PA groups.

Circadian clock abnormalities are linked not only to circadian rhythm disorders but also to a wide variety of common diseases, including hypertension, diabetes, obesity, and cancer [12]. Doi [6] found that disruption of the two cryptochrome genes Cry1 and Cry2 induces salt-dependent hypertension due to abnormally high aldosterone production by the adrenal gland. We did not find correlations between Cryptochrome and ARR in OSA patients with PA. We failed to replicate Doi’s findings in our OSA patients. Chronic intermittent hypoxia [9] seems to be the most plausible cause of hypertension in OSA yet can only partially explain the pathogenetic mechanism of the phenomenon. The hypertension of OSA patients is frequently severe and responds differently to drug therapy. We still cannot explain the mechanism of this phenomenon.

Neuroglobin (Ngb) is expressed in neurons of the central and peripheral nervous systems, and also the retina. Ngb expression is induced by neuronal hypoxia, cerebral ischemia and probably other pathophysiological factors, and that Ngb protects neurons subject to profound hypoxia or focal cerebral ischemia, and perhaps also neurodegenerative diseases [13–15]. Ngb is highly expressed in the rat suprachiasmatic nucleus (SCN) [1]. Because of its localization, it is surmised that Ngb plays a role in circadian rhythm. Our study has found this link, in OSA and PA Ngb correlates with Cry1 but not Cry2. Hundahl [3] found a link between Ngb and Per1, this study found that Ngb also affected Cry1.

In this study we found that Cry1 is more affected than Cry2, also Ngb correlated with Cry1 but not Cry2. Anand et al [16] found in their study that although both Cry proteins slowed the clock, Cry1 was significantly more potent than Cry2, and in SCN Cry1 but not Cry2 prolonged the interval of transcriptional suppression. Furthermore, Kovanen [17] found an association of Cry1 with arterial hypertension and elevated blood pressure. In this study we did not find correlation between Cry1 and PA, however all of our patients were hypertensive. One of the limitations of this study is the lack of control group with no hypertension.

We found significant relationship between lowest oxygen desaturation (nadir O_2_) and Cry1 expression in OSA subjects. We conclude that severe hypoxia in the setting of chronic intermittent hypoxia may cause decreased Cry1 expression. Yu et al and Egg et al had discovered that hypoxia can influence circadian rhythm [18,19]. In their study, Yu et al proved that hypoxia decreased Per1, Per2, Per3, and Cry1 mRNA expression [18]. Our study supported the finding that hypoxia may alter circadian function.

The strength of this study is the homogeneity of our subjects to better test our hypothesis. Limitations of this study are cross sectional design, small sample size, lack of a control group, and lack of 24-hour blood pressure monitoring. More studies should be done in order to look into the role of circadian rhythm especially Cry1 and Ngb in OSA and hypertension.

In conclusion, in this study we found Ngb correlates with Cry1 in OSA patients with PA. It is possible that hypoxia may alter biological rhythms. However, we still do not know what role Ngb plays in circadian rhythm. In this study we failed to prove correlations between Cry1 and Cry2 with PA. Ngb and Cry1 are down regulated in OSA with PA, although the mechanism of hypertension and PA is still unknown. Ngb is usually upregulated in hypoxic ischemic injuries, probably chronic intermittent hypoxia in OSA patients is not strong enough to increase Ngb and exert its neuroprotective capabilities in these patients.
